# Murine B Cell Development and Antibody Responses to Model Antigens Are Not Impaired in the Absence of the TNF Receptor GITR

**DOI:** 10.1371/journal.pone.0031632

**Published:** 2012-02-06

**Authors:** Lenka Sinik Teodorovic, Carlo Riccardi, Raul M. Torres, Roberta Pelanda

**Affiliations:** 1 Integrated Department of Immunology, National Jewish Health and University of Colorado Denver School of Medicine, Denver, Colorado, United States of America; 2 Department of Clinical and Experimental Medicine, University of Perugia, Perugia, Italy; University of Montreal, Canada

## Abstract

The Glucocorticoid-Induced Tumor necrosis factor Receptor GITR, a member of the tumor necrosis factor receptor superfamily, has been shown to be important in modulating immune responses in the context of T cell immunity. B lymphocytes also express GITR, but a role of GITR in humoral immunity has not been fully explored. To address this question, we performed studies to determine the kinetics of GITR expression on naïve and stimulated B cells and the capacity of B cells to develop and mount antibody responses in GITR^−/−^ mice. Results of our studies indicate that all mature B cells express GITR on the cell surface, albeit at different levels. Expression of GITR on naïve mature B cells is upregulated by BCR signaling, but is counteracted by helper T cell-related factors and other inflammatory signals *in vitro*. In line with these findings, expression of GITR on germinal center and memory B cells is lower than that on naïve B cells. However, the expression of GITR is strongly upregulated in plasma cells. Despite these differences in GITR expression, the absence of GITR has no effect on T cell-dependent and T cell-independent antibody responses to model antigens in GITR^−/−^ mice, or on B cell activation and proliferation *in vitro*. GITR deficiency manifests only with a slight reduction of mature B cell numbers and increased turnover of naïve B cells, suggesting that GITR slightly contributes to mature B cell homeostasis. Overall, our data indicate that GITR does not play a significant role in B cell development and antibody responses to T-dependent and independent model antigens within the context of a GITR-deficient genetic background.

## Introduction

Through the interplay of receptors and ligands, tumor necrosis factor superfamily (TNFSF) members play a role in numerous biological processes. By affecting cell signaling, survival, proliferation and/or differentiation, TNFSF members regulate the immune system resulting in either beneficial or harmful effects [1]. One such protein is TNFRSF18 or glucocorticoid-induced TNF receptor family-related gene (GITR), which shares many characteristics with some other TNFRSF members, namely OX40, 4-1BB and CD27 [2]. Like those TNFSF receptors, GITR only interacts with one ligand, GITRL [3], which is prevalently expressed by professional antigen presenting cells such as dendritic cells, macrophages and B cells [4], [5].

The function of GITR has been studied mainly in T cells, which express GITR constitutively. Regulatory T cells express the highest level of GITR. However, antigen receptor stimulation of naïve conventional T cells leads to GITR upregulation to levels observed in regulatory T cells [6], [7], [8], [9]. Studies in T cells suggest that GITR plays an important role in modulating T cell responses. Deletion of GITR, or use of reagents that block GITRL, prevent optimal proliferation of T cells in response to CD3 stimulation with or without CD28 co-stimulation [5], [8], suggesting that GITR may function to improve the magnitude of T cell responses. In fact, GITR is required for the survival of antigen-specific CD8 T cell clones and their expansion in response to influenza infection [10]. Moreover, stimulation of effector T cells through GITR provides them with resistance to suppression by regulatory T cells [5], while GITR stimulation of regulatory T cells inhibits their suppressive function [6], [7]. Thus, GITR operates in different ways to promote T cell responses. In fact, *in vivo*, GITR was shown to enhance immune responses to tumors and viral pathogens, but also contribute to the development of autoimmune diseases [7], [11], [12], [13], [14].

Hematopoietic cell types other than T cells, specifically macrophages and B cells, have been shown to express low levels of GITR [7]. However, the functional role of GITR in these cells has not been explored. In this study, we investigated the regulation of GITR expression on B cells and its potential function in B cell development and B cell response.

Our studies demonstrate that B cells express GITR starting at the transitional stage of B cell development. GITR expression is positively modulated by BCR signaling and negatively regulated by factors commonly provided by helper T cells. Despite the fact that all B cells express GITR, our data indicate that in the context of a whole GITR-deficient genetic background, this TNFSF receptor does not play a significant role in B cell development, B cell proliferation in response to antigen receptor stimulation and T cell help factors *in vitro*, and antibody responses to T cell-dependent and T cell-independent model antigens *in vivo*. The absence of GITR manifests only with a slight reduction of mature B cell numbers and increased B cell turnover, suggesting that GITR plays a small role in B cell homeostasis.

## Results

### B cell expression of GITR

**Figure 1:**
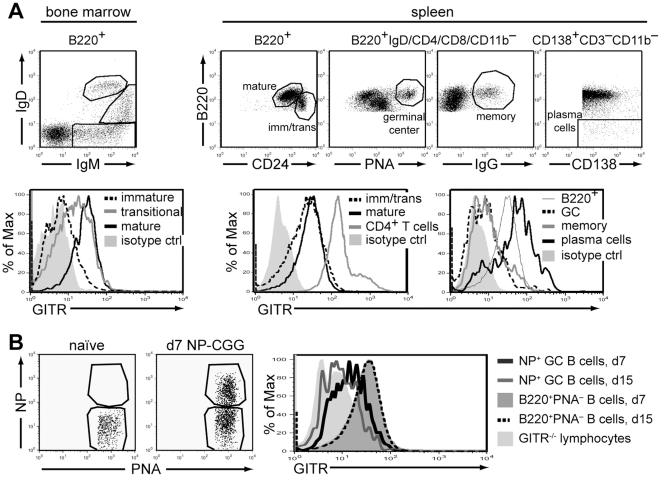
B cells express GITR starting at the transitional stage of development. (A) Bone marrow and spleen cells from wild-type (129S1) mice were analyzed *ex vivo* by flow cytometry for the expression of indicated markers. Live cells in dot plots were gated as indicated on top. Histograms depict GITR expression on B cells in subsets gated as shown in the dot plots in top panel. Expression of GITR on gated CD4^+^ T cells is shown as a positive control for GITR staining. Rat IgG2b was used as an isotype control for GITR staining. Data are representative of 3 independent experiments. Similar GITR expression was observed on B cells from BALB/c mice (data not shown). (B) GITR^+/+^ (BALB/c) mice were immunized i.p. with 100 µg NP_39_-CGG in Alum. At days 4, 7, and 15 post immunization, spleen cells were harvested from 2 immunized and 2 nonimmunized (naïve) mice at each point and analyzed for GITR expression on germinal center B cells reactive with NP. The dot plots show NP and PNA binding on B220^+^PNA^high^ gated B cells from one naive (left panel) and one immunized (right panel) mouse at day 7. There were no NP^+^ germinal center B cells at day 4, and reduced frequency at day 15 (data not shown). The histogram represents live, B220^+^IgD^−^CD3^−^CD11b^−^PNA^high^ germinal center B cells. GITR expression on NP^+^ germinal center B cells of one mouse at day 7 (black intact line) and one mouse at day 15 (gray intact line) following immunization are shown compared to those on total B220^+^PNA^−^ B cells (dark-shaded histogram and dashed black line) in respective animals and on lymphocytes of one GITR^−/−^ mouse (light-shaded histogram).

Previous studies reported low levels of GITR expression on mature B cells [7]. To determine when GITR is first expressed on developing B cells and its level on different B cell subsets, bone marrow and spleen B cells of wild-type mice were analyzed by flow cytometry for the expression of GITR and B cell developmental markers. In the bone marrow, some B220^+^IgM^+^IgD^−^ immature B cells displayed GITR slightly above antibody isotype control (Fig. 1A), while pro-B cells and pre-B cells had no detectable GITR expression (data not shown). GITR was expressed on all bone marrow IgM^high^IgD^low^ and spleen B220^+^CD24^high^ transitional B cells, although these cell populations displayed large variation in expression level (Fig. 1A). GITR expression was further increased, on average, on bone marrow recirculating IgM^low^IgD^high^ mature B cells, which displayed levels similar to those of splenic B220^+^CD24^low^ mature B cells (Fig. 1A). Despite the detection of GITR on naïve B cells, this level was considerably lower than that on naïve CD4^+^ T cells, which were used as positive control (Fig. 1A). GITR expression was also evaluated on antigen activated B cells (i.e., germinal center and memory B cells and plasma cells) that arise in response to environmental antigens in mice maintained in specific-pathogen free conditions (Fig. 1A). Compared to naïve mature B cells, germinal center (B220^+^IgD^−^PNA^+^) and memory (B220^+^IgG^+^) B cells exhibited lower levels of GITR that were similar to those of bone marrow immature B cells (Fig. 1A). Antigen-specific germinal center B cells that were analyzed 7 and 15 days following immunization with a T-dependent antigen also expressed GITR at lower levels than naïve B cells (Fig. 1B). Expression of GITR was upregulated on B220^−^CD138^+^ plasma cells, which expressed the highest level among the B cell subsets even when the difference in cell size was taken into account (Fig. 1A, and data not shown). Lymph node B cells expressed GITR at levels similar to those of splenic B cells (data not shown).

These data indicate that GITR is expressed constitutively on all B cells starting at the transitional B cell stage although its level varies during B cell development and within B cell subsets.

### BCR stimulation enhances GITR expression on B cells

**Figure 2:**
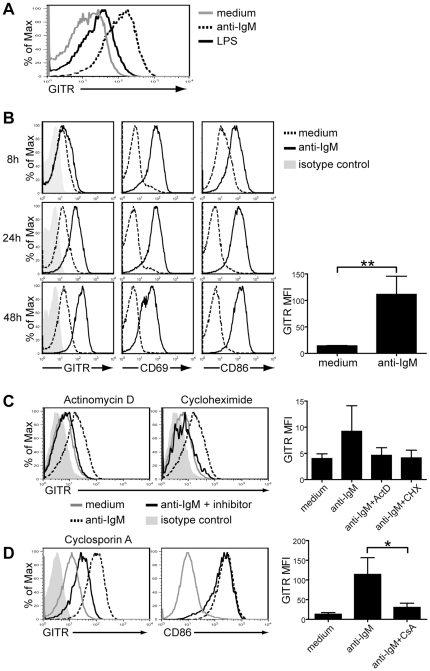
BCR stimulation enhances GITR expression on B cells. B cells were purified from the spleen of BALB/c mice by negative selection and all B cell cultures were performed in the presence of 4 ng/ml of BAFF to sustain cell survival. (A) Representative GITR expression on B cells cultured for 48 h in medium alone (gray line) or in the presence of either 10 µg/ml F(ab′)_2_ anti-IgM antibodies (dashed line) or 20 µg/ml LPS (intact black line). (B) B cells were cultured in either medium alone (dashed line) or with 10 µg/ml F(ab′)_2_ anti-IgM antibodies (intact line). Expression of GITR, CD69 and CD86 were analyzed at 8, 24 and 48 h of culture. The bar graph represents average GITR mean fluorescent intensity (MFI) on B cells cultured with medium or anti-IgM antibodies for 48 h from 3 independent experiments. **p≤0.002, *n* = 3. (C) B cells were stimulated in culture for 24 h with 10 µg/ml F(ab′)_2_ anti-IgM antibodies without (dashed line) or with 0.1 µM actinomycin D (ActD) to inhibit transcription, or 2 µM cycloheximide (Chx) to inhibit protein synthesis (intact black line). Gray line indicates GITR expression on cells cultured in medium alone. The diluent DMSO (0.2–0.4%) was present in all cultures. The bar graph represents average GITR MFI on B cells cultured with medium, anti-IgM antibodies, or anti-IgM antibodies with the indicated inhibitor from 3 independent experiments. Differences were not statistically significant, but were observed in all three independent experiments. (D) GITR (left) and CD86 (right) expression on B cells cultured for 48 h with 10 µg/ml F(ab′)_2_ anti-IgM antibodies alone (dashed line) or in the presence of 0.2 µM of cyclosporin A (CsA, intact black line). Expression on B cells cultured in medium alone (gray line) is shown for comparison. The bar graph represents average GITR MFI on B cells cultured with medium, anti-IgM antibodies, or anti-IgM antibodies with CsA from 3 independent experiments. *p≤0.05, *n* = 3. All histograms represent live, B220^+^ lymphoid cells. Isotype control staining for GITR (light shaded histogram) is shown in (B) and (C) and it was similar on stimulated or nonstimulated cells (data not shown). Data are representative of 2–6 independent experiments.

LPS was previously shown to induce GITR upregulation on B cells [7]. It is also known that T cells upregulate GITR upon CD3 stimulation [6], [7], [8], [9]. To test whether stimulation of B cells *via* the B cell receptor (BCR) could similarly induce GITR upregulation, we treated spleen B cells with an optimal concentration (10 µg/ml) of F(ab′)_2_ anti-IgM polyclonal antibodies for up to 72 h and analyzed GITR levels by flow cytometry at different time points. In fact, B cells stimulated *via* their BCR for 48 h upregulated GITR to a much greater extent, on average, than B cells stimulated with 20 µg/ml of LPS (Fig. 2A). GITR upregulation was initially observed on some B cells after 8 hours of BCR stimulation (Fig. 2B). By 24 hours, GITR was upregulated on average 5-fold on all B cells and reached maximum levels at 48 hours (Fig. 2B). CD69 and CD86, two well-described B cell activation markers, were analyzed alongside GITR to confirm B cell activation and compare the kinetics of upregulation. As shown in Fig. 2B, expression of CD69 and CD86 was already increased on all B cells after 8 hours of BCR stimulation, indicating that their upregulation preceded that of GITR. Conversely, GITR expression after 72 hours of BCR stimulation was maintained at levels comparable to those at 48 hours while CD69 was downmodulated (data not shown).

These data indicate that BCR stimulation of B cells, similar to TCR stimulation of T cells, promotes higher expression of GITR, which is sustained for at least 72 hours.

### Increased expression of GITR on BCR-stimulated B cells is due to *de novo* transcription and translation, and is partly dependent on NFAT signaling

To assess whether the increase in GITR levels mediated by BCR stimulation on B cells was due to either *de novo* synthesis or the presence of pre-formed pools of GITR, we used actinomycin D and cycloheximide to inhibit gene transcription and translation, respectively. For these studies, B cells were evaluated after 24 hours of culture because the transcription/translation inhibitors caused extensive cell death at a later time. A drawback of the short time cultures, however, was that GITR was not upregulated to the maximum level (Fig. 2B). Additionally, we found that DMSO, used as a diluent for the inhibitors and added to control cultures, inhibited somewhat GITR expression on B cells. Nevertheless, results of these studies show that addition of either actinomycin D or cycloheximide during BCR stimulation of B cells for 24 hours prevented the upregulation of GITR (Fig. 2C). Due to the variability in GITR expression on BCR-stimulated B cells, this difference was not statistically significant (*p* = 0.07), but inhibition of GITR upregulation was reproducibly observed in all experiments (*n = 3*). Importantly, actinomycin D and cyclohexamide did not alter upregulation of CD69 on BCR-stimulated B cells (data not shown), similarly to what was previously observed in T cells [15], indicating that inhibition of GITR upregulation was not due to cell stress or death. These data indicate that GITR upregulation necessitates *de novo* gene transcription and translation, potentially explaining why it requires 8–24 hours for higher GITR expression to ensue.

Since BCR signaling modulates GITR expression, we wanted to assess which downstream signaling pathway might be involved in GITR regulation. Zhan *et al.* [9] has reported that GITR expression is modulated by the NFAT signaling pathway in T cells. To determine whether NFAT regulates GITR expression on B cells, BCR-stimulated spleen B cells were treated with cyclosporin A (CsA), an NFAT pathway inhibitor. Treatment of B cells with CsA prevented maximum upregulation of GITR on BCR-stimulated B cells and this difference was statistically significant (Fig. 2D). CD86 levels, in contrast, were not affected by CsA (Fig. 2D), indicating intact cell function. Our data, therefore, suggest that NFAT signaling promotes GITR upregulation on BCR-stimulated B cells.

### Helper T cell factors inhibit GITR upregulation induced by BCR signaling on B cells

**Figure 3:**
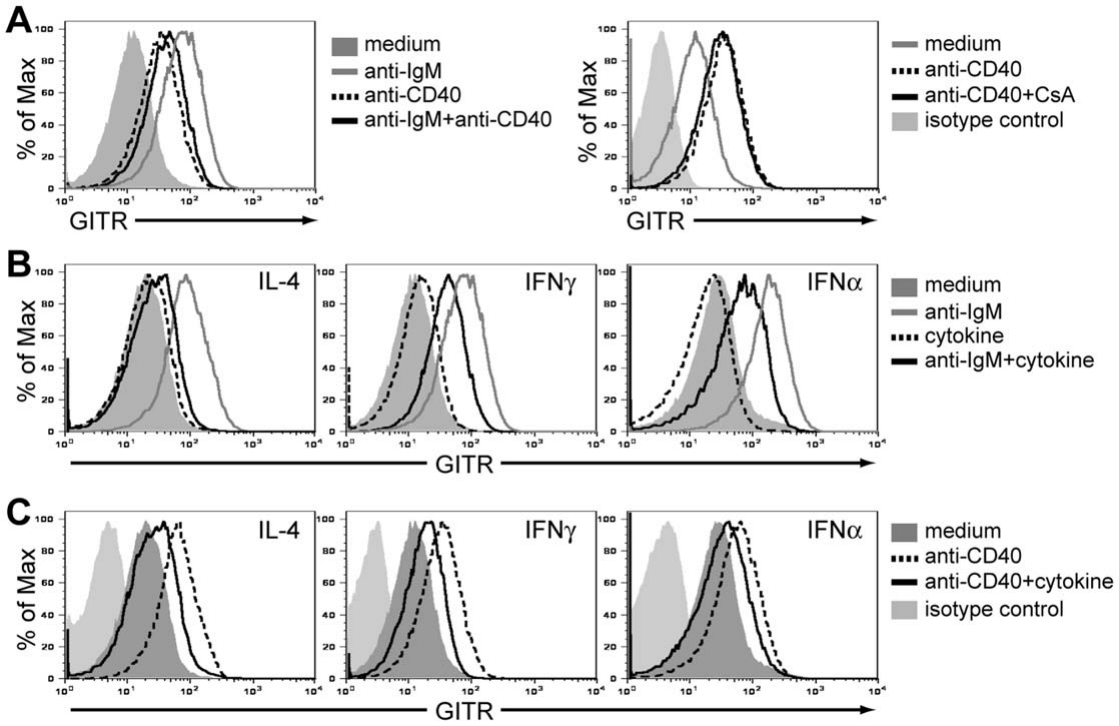
Helper T cell factors inhibit GITR induction on BCR-stimulated B cells. B cells were purified from the spleen of wild-type BALB/c mice by negative selection and used for *in vitro* analyses. All B cell cultures were performed in the presence of 4 ng/ml of BAFF to sustain cell survival. (A) GITR expression on B cells cultured for 48 h in medium alone, with 2 µg/ml F(ab′)_2_ anti-IgM antibodies (gray line, left panel), 15 µg/ml anti-CD40 antibodies (dashed line, left and right panels), a combination of anti-IgM and anti-CD40 antibodies (intact black line, left panel), or a combination of anti-CD40 antibodies and 0.2 µM CsA (intact black line, right panel). (B) GITR expression on B cells cultured for 48 h in medium alone (filled histogram), 2 µg/ml F(ab′)_2_ anti-IgM antibodies (gray line), 50 ng/ml IL-4, 100 (not shown) and 1000 U/ml IFNγ, 100 (not shown) and 1000 U/ml IFNα (dashed line) or a combination of anti-IgM antibodies and cytokines (intact black line) as indicated. There was no difference between 100 and 1000 U/ml of IFNγ and IFNα (data not shown). Isotype control staining for GITR (light shaded histogram) is shown in (A) and (B) and it was similar on stimulated or nonstimulated cells (data not shown). (C) GITR expression on B cells cultured with 15 µg/ml anti-CD40 antibodies alone (dashed line) or in combination with 50 ng/ml IL-4, 1000 U/ml IFNγ, or 1000 U/ml IFNα (intact line), as indicated. Note that the isotype control antibody staining was included in all analyses but omitted in plots that display more than 3 samples for clarity.

The upregulation of GITR expression mediated by BCR signaling on naïve B cells contrasted the observation that germinal center and memory B cells, which are antigen-activated cells, expressed GITR at levels lower than those of naïve B cells (Fig. 1). Given these contrasting findings, we next explored how GITR levels are modulated by T cell factors, with the hypothesis that helper T cell-specific factors might decrease GITR expression. To this end, we utilized anti-CD40 antibodies and/or recombinant cytokines that recapitulate signals normally provided by T cells and other cells during Th1 and Th2 responses. Hence, 15 µg/ml of anti-CD40 antibodies, 50 ng/ml of IL-4, 100 or 1000 U/ml of IFNγ or 1000 U/ml of IFNα were added to B cell cultures, with or without BCR stimulation (Fig. 3 and data not shown). This level of CD40 stimulation on B cells increased GITR expression albeit to a lower level than that induced by BCR stimulation (Fig. 3A, left panel). The induction of GITR expression by CD40 was not mediated *via* the NFAT pathway, as the addition of CsA to anti-CD40-treated B cells did not prevent GITR upregulation (Fig. 3A, right panel). In contrast to the positive effect of anti-CD40 on GITR expression, IL-4, IFNγ, or IFNα alone did not alter GITR levels on B cells after 48 hours of treatment (Fig. 3B). Interestingly, when anti-CD40 antibodies, IL-4, IFNγ, or IFNα were added to B cells in combination with BCR (anti-IgM) stimulation, these signals inhibited maximum GITR upregulation compared to BCR stimulation alone (Fig. 3B). Thus, our results indicate that some helper T cell components, at least at the doses used here, block maximum GITR upregulation observed in response to a BCR signal on B cells. This effect was unique to GITR, as CD69 and CD86 levels increased as expected (data not shown). Moreover, IL-4, IFNγ, and IFNα were also able to inhibit GITR upregulation mediated by CD40 stimulation on B cells (Fig. 3C). These findings may explain the observation that germinal center and memory B cells express low levels of GITR, as these B cell subsets are the result of a cognate B cell-T cell interaction.

These data suggest that while GITR is upregulated by BCR signaling and may function on B cells receiving signal 1 (antigen binding), the expression and signaling of this TNF family receptor on B cells may be negligible in the context of a T-dependent response.

### B cell development in GITR-deficient mice

**Figure 4:**
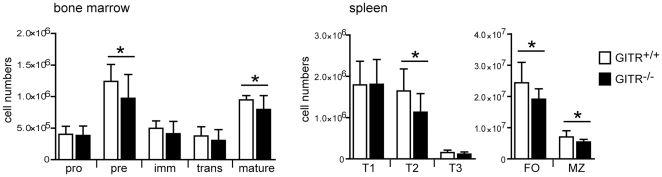
Absence of GITR expression slightly affects generation or maintenance of mature B cells. Bone marrow and spleen cells from GITR^+/+^ (129S1 strain, white bars) and GITR^−/−^ (black bars) mice were analyzed *ex vivo* by flow cytometry to determine the numbers of B cells at different developmental stages. The following B220^+^ B cell subsets were defined in the bone marrow: pro-B (IgM^−^CD2^−^), pre-B (IgM^−^CD2^+^), immature B (IgM^+^IgD^−^), transitional B (IgM^high^IgD^low^) and mature/recirculating B (IgM^low^IgD^high^) cells. The following B220^+^ B cell subsets were defined in the spleen: transitional T1 (CD93^+^IgM^+^CD23^−^), transitional T2 (CD93^+^IgM^+^CD23^+^), transitional T3 (CD93^+^IgM^low^CD23^+^), follicular (FO, CD1d^−/low^CD21^+^), and marginal zone (MZ, CD1d^high^CD21^high^). *n* = 10 from 5 independent experiments. Error bars represent SD; *p<0.05.

The differential expression of GITR by B cell subsets and its regulation in the context of BCR and cytokine receptor stimulation suggest a potential role of GITR during B cell development and/or function. To begin to evaluate GITR function in B cells, we examined whether the absence of GITR affects B cell development. Thus, we enumerated B cells at all stages of development from pro-B cells to naïve mature B cells in GITR^−/−^ [16] and control (129S1) mice. There was a small but significant reduction of pre-B cells in GITR^−/−^ mice, which did not significantly affect immature and transitional T1 B cell numbers (Fig. 4). Nonetheless, GITR^−/−^ mice displayed a significant, albeit small, reduction of transitional T2 and mature B cell numbers (Fig. 4). In particular, all mature B cell subsets analyzed, including the recirculating B cell population in the bone marrow and the follicular and marginal zone B cell subsets of the spleen, were similarly decreased in GITR^−/−^ mice relative to control animals (Fig. 4). Thus, while GITR is not required for early B cell development, it appears to play a small role in the generation and/or maintenance of mature B cells.

### GITR expression and signaling are not necessary for B cell costimulation and proliferation *in vitro*

**Figure 5:**
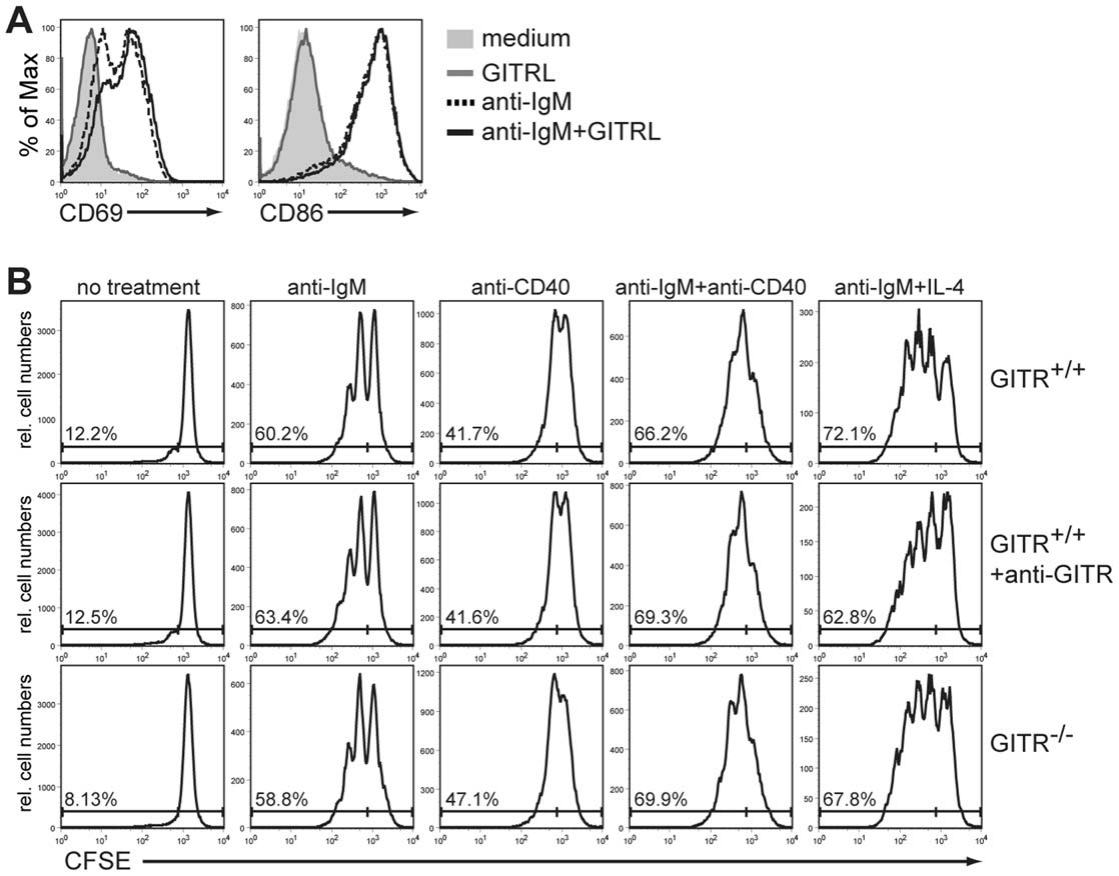
GITR expression and signaling do not alter B cell activation and proliferation. (A) B cells were purified from the spleen of wild-type BALB/c mice by negative selection and cultured for 48 h in medium alone (filled histogram) or in the presence of 1 µg/ml GITRL (gray line), 2 µg/ml F(ab′)_2_ anti-IgM antibodies (dashed line) or a combination of these reagents (intact black line). All B cell cultures were performed in the presence of 4 ng/ml of BAFF to sustain cell survival. Histograms indicate CD69 (left) and CD86 (right) expression on live B220^+^ lymphocytes. Data are representative of 3 independent experiments. (B) Total spleen cells from GITR^+/+^ (129S1 strain) and GITR^−/−^ mice were purified over a Ficoll gradient, labeled with CFSE and cultured for 4 days. Cultured cells were either left untreated or stimulated with 10 µg/ml F(ab′)_2_ anti-IgM antibodies, 15 µg/ml anti-CD40 antibodies, a combination of anti-IgM and anti-CD40 antibodies, or a combination of anti-IgM antibodies and 50 ng/ml IL-4, as indicated at the top. All B cell cultures were performed in the presence of 4 ng/ml of BAFF to sustain cell survival. Cells from GITR-sufficient mice were treated as stated above in the presence or absence of 10 µg/ml anti-GITR agonistic antibodies (DTA-1 clone). Histograms represent CFSE dilution in live, B220^+^ B lymphocytes. Numbers represent frequency of dividing (CFSE^low^) cells in the population. Data are representative of 2 independent experiments.

To further assess the role of GITR in B cell function, we asked whether GITR acts as a costimulatory molecule during B cell activation. To this end, splenic B cells were stimulated *in vitro* with a suboptimal concentration (2 µg/ml) of anti-IgM antibodies in the presence or absence of recombinant GITR ligand (GITRL). The functionality of the GITRL reagent was confirmed by its ability to increase proliferation of wild-type, but not GITR^−/−^, CD4^+^ T cells (data not shown). A suboptimal concentration of anti-IgM was used to ensure that the BCR signaling alone did not override possible effects induced by GITR signaling. B cell activation was evaluated by the expression of the activation markers CD69 and CD86. Our results show that addition of GITRL to B cells, whether alone or with anti-IgM antibodies, did not lead to increased expression of CD69 and CD86 (Fig. 5A). Similar results were obtained using different concentrations (0.1, 0.5 or 10 µg/ml) of anti-IgM antibodies (data not shown).

Previous studies have shown that GITR is important for the proliferation of T cells in response to CD3 stimulation [5], [8], [16]. To test if GITR also affects B cell proliferation, we labeled total spleen cells from GITR^−/−^ and control mice with CFSE and cultured them for four days with or without BCR and co-receptor stimulation. We reasoned that some spleen cells expressed GITRL in these cultures, providing signaling function to GITR on B cells, although we were unable to detect GITRL expression using commercial and non-commercial antibodies (data not shown). Therefore, to further ensure GITR signaling, agonistic anti-GITR antibodies [7] were added to some cell cultures. GITR-deficient B cells proliferated to the same extent as wild-type B cells when treated with 10 µg/ml anti-IgM and/or 15 µg/ml anti-CD40 antibodies, or with anti-IgM together with IL-4 (Fig. 5B, top and bottom panels). Additionally, stimulation of wild-type B cells with agonistic anti-GITR antibodies did not aid in cell proliferation (Fig. 5B, middle panel). Finally, no significant differences in B cell survival were observed in GITR^−/−^ B cells (data not shown).

Overall, these data indicate that GITR does not function as a co-stimulatory receptor in B cell activation and proliferation.

### GITR-deficient mice respond normally to T cell-dependent and T cell-independent model antigens *in vivo*

**Figure 6:**
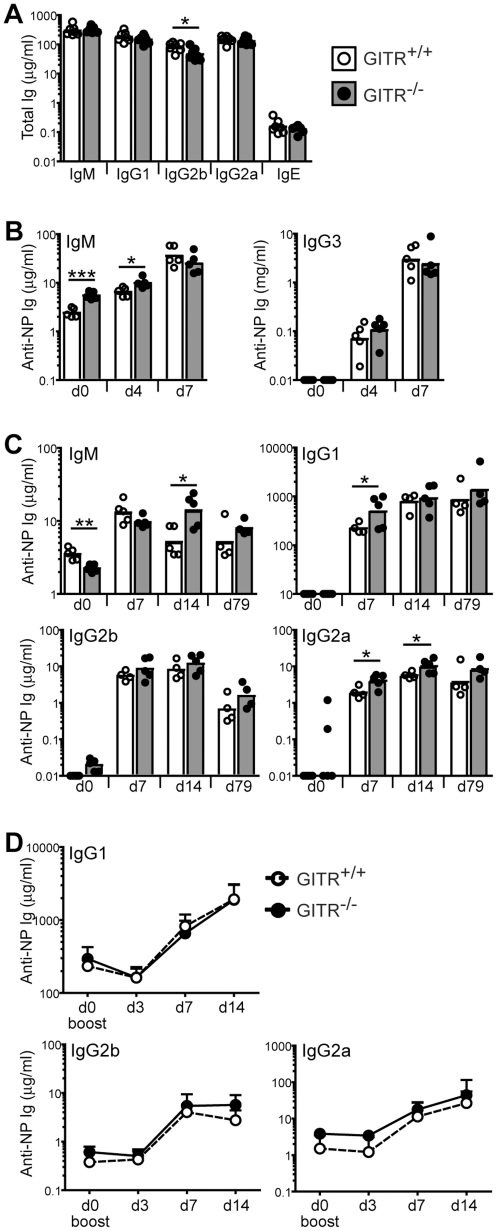
GITR-deficient mice mount relatively normal antibody responses to model antigens. (A) Total serum levels of IgM, IgG1, IgG2b, IgG2a and IgE in 4 month-old GITR^+/+^ (129S1 strain, white bars, empty circles) and GITR^−/−^ mice (gray bars, filled circles). *n* = 7. (B) GITR^+/+^ (129S1, white bars, empty circles) and GITR^−/−^ (grey bars, filled circles) mice (*n* = 5 per group) were immunized i.p. with 5 µg NP_28_-FICOLL. Sera were collected before (day 0) and 4 and 7 days after immunization, and NP-specific IgM and IgG3 were measured by ELISA. (C) GITR^+/+^ (129S1, white bars, empty circles) and GITR^−/−^ (grey bars, filled circles) mice (*n* = 5 per group) were immunized i.p. with 100 µg NP_36_-CGG in Alum. Sera were collected at indicated time points and NP-specific IgM, IgG1, IgG2b and IgG2a were measured by ELISA. (D) Mice described in (C) were boosted with 5 µg/ml NP_36_-CGG injected i.p. 178 days after primary immunization, and sera were collected at indicated times after boost. NP-specific IgG1, IgG2b and IgG2a were measured by ELISA (*n* = 4 per group). Bars and circles represent geometric means and individual mice, respectively. *p<0.05; **p≤0.002; ***p≤0.0002.

Antibody production is one of the main functions of B cells. We tested whether GITR affects antibody production by measuring first total levels of Ig isotypes in GITR^−/−^ and control mice. As shown in Fig. 6A, total levels of IgM, IgG1, IgG2a and IgE were normal in GITR^−/−^ mice, despite a slight reduction of mature B cell numbers (Fig. 4), while those of IgG2b were slightly depressed.

Since BCR signaling and T cell-like help modulated GITR levels on B cells, we next tested whether GITR affects B cell responses to T-independent (TI) and T-dependent (TD) antigens. To examine TI responses, mice were immunized with a model TI type 2 antigen, the hapten NP conjugated to Ficoll (NP-Ficoll). Sera were collected prior to immunization and at days 4 and 7 after immunization to measure NP-specific IgM and IgG3, the predominant isotypes produced in response to this antigen. Pre-immune and day 4 NP-specific IgM levels were slightly higher in GITR^−/−^ mice compared to wild-type levels (*p* = 0.0002 and 0.01, respectively), while day 7 levels were comparable between the two groups (Fig. 6B). NP-specific IgG3 levels were similar at all time points measured. Thus, GITR-deficient mice mount a relatively normal response to NP-Ficoll immunization.

To evaluate the role of GITR in T-dependent antibody responses, GITR^−/−^ and control mice were immunized with a TD model antigen, NP conjugated to chicken gamma globulin (NP-CGG) in Alum, which is known to induce predominantly the IgG1 isotype. NP-specific IgM, IgG1, and IgG2a levels were slightly higher in GITR^−/−^ mice at 7 and/or 14 days of the immune response, while IgG2b levels were similar in both groups (Fig. 6C). Moreover, immunization of mice with a combination of NP-CGG and poly(I∶C) resulted in similar NP-specific (and CGG-specific) IgM, IgG2b and IgG2a, and lower IgG1 antibody responses in GITR-deficient relative to GITR-sufficient mice (data not shown), suggesting that the small differences observed following NP-CGG/Alum immunization were not reproducible. Additionally, antibody differences observed during NP-CGG/Alum response were not maintained over time, as 79 days after immunization the mice exhibited similar levels of NP-specific Ig isotypes.

The amount of high affinity NP-specific IgG1 antibodies in GITR^−/−^ mice at day 14 of the TD-response, as measured on ELISA plates coated with low avidity antigen, was also normal (data not shown), indicating normal affinity maturation at this time point. Our results, therefore, indicate that GITR-deficient mice mount a normal primary immune response to TI and TD model antigens.

To study the effect of GITR on secondary TD responses, mice that were previously immunized with NP-CGG in Alum, were boosted with a second NP-CGG immunization in the absence of adjuvant 178 days after the primary immunization. NP-specific IgG1, IgG2b and IgG2a levels were measured at 3, 7 and 14 days after boosting. The memory response was comparable between GITR-deficient and GITR-sufficient B cells (Fig. 6D).

Overall, these data indicate that GITR is not required for TI and TD antibody responses to the model NP antigens. Furthermore, GITR does not affect memory antibody responses to the TD antigen NP-CGG.

### GITR-deficient B cells display normal abilities in entering the antigen-selected B cell populations in GITR^−/−^ mice

**Figure 7:**
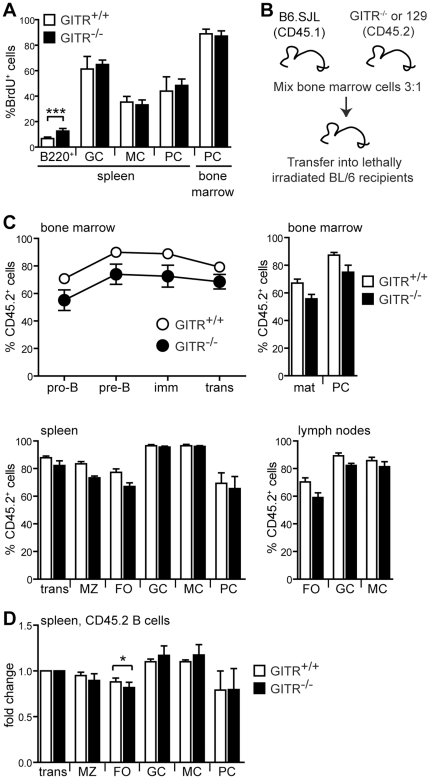
GITR appears dispensable for entry of B cells in the active cell subsets. (A) GITR^+/+^ (129S1, white bars) and GITR^−/−^ (black bars) mice (*n* = 5) were fed BrdU continuously for one week. Bars represent frequency of BrdU^+^ cells in naïve B220^+^ (PNA^−^), germinal center (GC), memory (MC), and plasma (PC) cell compartments gated as shown in Fig. 1. Error bars represent SD. ***p≤0.003. Similar results were found in mice fed BrdU for two weeks (data not shown). (B) Schematic for the generation of GITR-deficient and sufficient mixed bone marrow chimeras. Bone marrow cells from CD45.2 GITR^−/−^ and GITR^+/+^ 129S1 mice were mixed at a 1∶3 ratio with bone marrow cells isolated from CD45.1 C57BL/6 SJL mice. Cell mixtures were injected i.v. into lethally irradiated C57BL/6 mice, which were evaluated 8 weeks later. (C) Bone marrow, spleen and lymph nodes from mixed bone marrow chimeras (*n* = 5 per group) generated as described in (B) were analyzed by flow cytometry to determine the frequency of CD45.2^+^ cells in B cell subsets. B cell subsets were gated as described in Figs. 1 and 4. Open bars and symbols represent CD45.2^+^ GITR^+/+^ B cells and filled bars and symbols represent CD45.2^+^ GITR^−/−^ B cells. The graphs represent means and SEM for each indicated B cell subset. (D) The relative size of the CD45.2^+^ B cell population in each spleen B cell compartment was measured relative to that of the transitional B cell population in each mouse described in (C). The bar graph represents the average fold change (+SEM) in the size of each CD45.2 B cell subset relative to the transitional B cell subset, which was set at 1. Open bars represent CD45.2^+^ GITR^+/+^ B cells and filled bars represent CD45.2^+^ GITR^−/−^ B cells. *p<0.05.

When naïve mature B cells undergo a productive antigen response, they undergo further differentiation into antigen-selected B cell subsets, such as germinal center and memory B cells and plasma cells. To determine whether GITR affects the antigen-mediated differentiation of B cells, groups of GITR^−/−^ and control mice were fed BrdU in drinking water. The frequency of BrdU^+^ cells in the germinal center, memory and plasma cell populations were measured after one or two weeks of BrdU treatment. Since antigen-mediated activation of B cells promotes cell proliferation and, therefore, BrdU incorporation, this methodology can be used to determine the kinetics of antigen-selected B cells *in vivo* [17]. One week after BrdU treatment, the frequency of BrdU^+^ cells in the germinal center, memory, and plasma cell populations was 40% to 90%, depending on the population (Fig. 7A). This high frequency reflected the fact that the entry of B cells into these subsets requires cell proliferation. In fact, the frequency of BrdU^+^ B cells in the naïve B220^+^ B cell population was only approximately 5–10% (Fig. 7A), in line with previous reports [18], [19], [20]. GITR^−/−^ and control mice displayed the same frequency of BrdU^+^ cells in the germinal center, memory, and plasma cell populations of the spleen, and plasma cell population of the bone marrow (Fig. 7A). Data collected after two weeks of BrdU feeding were similar to those at the end of week one (data not shown). These data suggest that antigen-selection and terminal differentiation of B cells are not affected in mice lacking GITR. However, the frequency of BrdU^+^ cells in the naïve B220^+^ B cell compartment was significantly higher in GITR^−/−^ than in control mice (Fig. 7A), indicating a higher turnover of naïve B cells in the absence of GITR, and correlating with reduced numbers of mature B cells (Fig. 4).

If GITR function in B cells is subtle, it might be difficult to note differences in intact GITR^−/−^ mice where all cells lack GITR expression. To address this issue, we tested how GITR-deficient B cells would function in competition with GITR-sufficient cells. To this end, we generated mixed bone marrow chimeras with GITR^−/−^ and GITR^+/+^ cells congenic at the CD45 locus (Fig. 7B). Bone marrow cells from GITR-deficient mice (GITR^−/−^, CD45.2) were mixed with B6.SJL bone marrow cells (GITR^+/+^, CD45.1) and injected into lethally irradiated B6 recipients. Control chimeras received a mix of bone marrow cells from wild-type 129S1 mice (GITR^+/+^, CD45.2) and B6.SJL. Bone marrow, spleen and lymph nodes were analyzed by flow cytometry to determine the size of CD45.2 B cell subsets, from the pro-B cell to the plasma cell stages of B cell differentiation (Fig. 7C). Bone marrow cells were mixed at a 3∶1 ratio favoring B6.SJL cells because seeding and growth of 129S1 hematopoietic stem cells is superior to that of B6 cells. Nevertheless, 129S1 hematopoietic cells still dominated the recipient mice. In addition, the frequency of CD45.2 cells was slightly higher at the beginning of B cell development (pro-B cells) in GITR^+/+^ than in GITR^−/−^ chimeras. Furthermore, the frequency of CD45.2 cells was not perfectly maintained during B cell differentiation, making it difficult to compare the two groups of mice. In spite of these issues, the frequency of CD45.2 B cells appeared to follow a similar pattern in both groups of chimeras (Fig. 7C). To better compare the two groups, we measured the fold changes of the CD45.2 B cell frequency in mature and antigen-selected B cell populations of the spleen relative to that of the immature/transitional (CD24^high^) B cell population in each mouse within the two groups (Fig. 7D). The only significant difference was in the follicular B cell population, which was slightly decreased in GITR^−/−^ chimeras (Fig. 7D). This result, therefore, was in line with the reduced follicular B cell population observed in intact GITR-deficient mice (Fig. 4) and the increased BrdU incorporation in B220^+^ cells (Fig. 7A).

Together, these data indicate that the absence of GITR does not hinder early B cell development, but it causes a small defect in the size of the follicular naïve B cell compartment. This defect does not affect further differentiation of B cells into antigen-selected B cell subsets, such as memory B cells and plasma cells.

## Discussion

This study was performed to determine whether the TNF family receptor GITR plays a role in basic B cell functions. We found that expression of GITR on mature B cells is upregulated by BCR signaling and downregulated by factors commonly provided by helper T cells. In spite of this, the absence of GITR had only minimal effects on mature B cell numbers, while it did not affect antibody responses to model antigens, or B cell activation and proliferation. These findings indicate that GITR does not play a significant role in B cell development and antibody responses in GITR-deficient mice.

We show that GITR is expressed at low levels on the surface of resting mature B cells, approximately 10-fold lower than those on naïve CD4 T cells. BCR stimulation, however, increases GITR expression on mature B cells by at least 10-fold within 24 hours. Thus, on BCR-stimulated B cells, GITR reaches levels comparable to those of naïve CD4 T cells. Like BCR stimulation on B cells, TCR stimulation on T cells also leads to upregulation of GITR by approximately 10-fold [6], [7], [8], [9]. Therefore, it appears that a common signaling pathway downstream of antigen receptors in both B and T cells leads to upregulation of GITR. Based on our experiments involving transcription and translation inhibitors, we conclude that the increase in GITR surface levels on BCR-stimulated B cells results from *de novo* gene transcription, which is perhaps the reason why maximum surface GITR levels are achieved by 24–72 hours, and not by 8 hours following antigen receptor stimulation.

In T cells, GITR upregulation was shown to be inhibited by NFAT signaling [9]. In contrast, we found that in B cells, inhibition of the NFAT pathway by CsA prevented maximum GITR upregulation following BCR stimulation. This suggests that NFAT promotes, and does not inhibit, GITR expression in B cells. We explain this difference by the fact that Zhan *et al.* determined the effect of NFAT on GITR expression on T cells stimulated with ionomycin, and not *via* the TCR [9]. In fact, when we analyzed GITR expression on CD3-stimulated T cells in the presence of CsA, we found that GITR upregulation required NFAT signaling (data not shown). In contrast, upregulation of GITR on T cells stimulated with ionomycin was increased by CsA as previously reported [9], but only when a low ionomycin concentration (200 ng/ml) was used (data not shown). NFAT inhibition was unable to completely suppress GITR expression on BCR-stimulated B cells, suggesting that additional molecular pathways control *Tnfrsf18* gene transcription. Candidate pathways may be those signaling *via* NF-κB and JNK, which have been additionally suggested to promote GITR expression in T cells [9].

Despite the fact that BCR signaling promotes GITR upregulation on B cells, neither GITR stimulation nor GITR-deficiency modulated expression of activation markers and proliferation of B cells stimulated with anti-IgM antibodies and additional stimuli. These findings suggest that GITR does not function as a costimulatory molecule for BCR signaling. GITR-deficient B cells in intact GITR^−/−^ mice and in mixed bone marrow chimeras displayed only a minor but consistent defect at the naïve mature cell stage. Namely, we observed slight, but significant, reduced numbers of GITR-deficient mature B cells. Furthermore, we observed a higher frequency of BrdU^+^ resting B cells in the spleen of GITR^−/−^ mice relative to control mice fed BrdU for one or two weeks. A higher frequency of BrdU^+^ B cells entering the peripheral mature B cell population within this timeframe suggests a reduced lifespan and an increased cell turnover. These findings support the idea that GITR slightly contributes to the survival of naïve mature B cells and to their homeostasis. A similar role of GITR on CD4^+^CD25^+^ T cell homeostasis and the survival of antigen activated CD8 T cells have been previously reported [5], [10].

GITR deficiency did not translate into defects in antibody responses to T-dependent and T-independent model antigens and entry of B cells into the antigen-activated cell populations. If GITR were important for the survival of antigen activated B cells, we expected to find a reduced BrdU incorporation in the B cell memory and plasma cell populations, a result we did not observe. A potential explanation for the lack of effects of GITR deficiency on B cell responses might be that GITR is not upregulated (or perhaps is downregulated) on activated B cells *in vivo* in the presence of various inflammatory stimuli. In fact, GITR was minimally expressed on antigen-activated B cells that had been exposed to helper T cell factors and inflammatory cytokines *in vitro*, and it was also poorly expressed on germinal center and memory B cells *ex-vivo*. Thus, activated B cells in the context of an intact immune system may be undistinguishable from GITR-deficient B cells in relation to GITR expression and, therefore, response. It has been reported that GITR stimulation greatly improves humoral responses to ovalbumin and hemagglutinin *in vivo*, potentially by increasing Th1 and Th2 cytokine production by T cells [21]. Based on this latter study, we anticipated finding a reduced antibody response to NP-CGG in GITR-deficient mice, a result we did not observe. These apparently discordant results may indicate that the role of GITR in immune responses depends on the type of antigen and adjuvant. Alternatively, the effect of GITR deficiency on B cells might be masked by its absence on other cell types in GITR-deficient mice (as observed for CD8 T cells, [10]) or might manifest only in certain genetic backgrounds. Such issues could be addressed by analyzing bone marrow chimeras in which only B cells lack GITR and by backcrossing GITR-deficient mice into other genetic backgrounds, respectively.

Another possible explanation for the minimal effects of GITR deficiency on B cells is that the function of GITR is shared by other TNF-family receptors [13]. Although our studies cannot exclude the possibility that other TNF-family receptors share a redundant function with GITR in B cells, we think that this is probably not the case. The reason is that when we evaluated two of the TNF-family receptors that are the most similar to GITR, CD27 and OX40, we found that they were not significantly expressed on resting B cells (data not shown). More importantly, expression of CD27 and OX40 was not significantly upregulated on either GITR-sufficient or GITR-deficient B cells upon BCR stimulation (data not shown), suggesting that CD27 and OX40 do not share a redundant function with GITR in B cells.

What other role, if any, may GITR play in B cells? The fact that expression of GITR on B cells is positively regulated by BCR stimulation and negatively regulated by factors provided by helper T cells suggests that GITR may function to discriminate the response of B cells that receive signal 1 (antigen) in the absence of signal 2 (e.g., T cell help, inflammatory cytokines) from that of B cells that receive both signals. For instance, GITR may be important in the context of B cell tolerance to self-antigens. Presently, this seems unlikely given that we were unable to detect anti-chromatin antibodies (the most common autoantibodies expressed when B cell tolerance is defective) in young and old GITR^−/−^ mice (data not shown). Alternatively, GITR might play a role in B cell responses that we have not tested such as cytokine production during immune responses, and generation of neutralizing antibodies during infections. Although not having a significant role in common B cell functions, GITR may still function as a marker to distinguish B cell subsets given that its expression decreases in GC and memory B cells while it increases in plasma cells relative to naïve B cells.

In conclusion, our findings indicate that, in the context of a whole GITR^−/−^ genetic background, GITR does not play a significant role in B cell generation or antibody responses to model antigens, but it slightly contributes to mature B cell homeostasis. Our data, therefore, support the idea that defects in immune responses observed in mice that lack GITR are not likely caused by changes in B cell function.

## Materials and Methods

### Mice

CB17, BALB/c, 129S1/svImJ (129S1), and B6.SJL mice were purchased from Jackson Laboratories and then bred or maintained in our facility. GITR-deficient (GITR^−/−^) mice [16] were imported from Italy and then bred in our facility. GITR^−/−^ mice were received and maintained on a 129SvJ genetic background. GITR^−/−^ mice were also bred to 129S1 in our facility to generate F1 mice that were then intercrossed to obtain F2 GITR^−/−^ mice. No differences were observed between the newly derived GITR^−/−^ mice and GITR^−/−^ mice derived from the original stock, indicating no effect of genetic background. Both females and males were used for experiments, and mice used for individual experiments were matched for age and sex. All animals were bred and maintained in specific pathogen-free rooms at the Biological Research Center at National Jewish Health, Denver, CO. All animal protocols were approved by the Institutional Animal Care and Use Committee (IACUC) under permit #A3026-01.

### Flow Cytometry and antibodies

Bone marrow and spleen single cells were resuspended in PBS, 3% FBS and stained with fluorochrome-conjugated or biotinylated antibodies against B220 (RA3-6B2), IgD (11-26c-2a), IgM^a^ (DS-1), CD2 (RM2-5), CD24 (M1/69), CD3 (145-2C11), CD4 (GK1.5), CD8 (53-6.7), CD11b (M1/70), CD138 (281-2), IgG1 (A85-1), IgG2a/2b (R2-40), CD93 (AA4.1), GITR (DTA-1), CD1d (1B1), CD21 (7G6), CD23 (B3B4), CD69 (H1.2F3), CD86 (GL1) (all from eBioscience or BD Pharmingen), CD19 (1D3), CD21 (7G6) IgM (R33-24.12) and IgD (1.3–5) (generated in house). Rat IgG2b (eBioscience) was used as isotype control for GITR staining. PNA (FL-1071; Vector Laboratories) was used for staining germinal center B cells. Biotin-labeled antibodies were visualized with fluorochrome-conjugated streptavidin (eBioscience). Propidium iodide (Sigma-Aldrich) was used is some experiments to exclude dead cells. Data acquisition was done on a CyAn (Beckman Coulter), Facscan (BD) or Facscalibur (BD) flow cytometer and analyzed with FlowJo software (Tree Star). Before each analysis, the flow cytometer was set using single color staining of each antibody on a mix spleen cell population that contained negative and positive cells. Cell analyses were performed on a doublet cell excluded (single), live and lymphoid (based on forward and side scatter) cell gate.

### *In vitro* cell culture and cell proliferation assay

Splenic B cells were purified by negative selection using anti-CD43 monoclonal antibody coupled to magnetic beads (Miltenyi Biotech) and an AutoMACS (Miltenyi Biotech) according to manufacturer's instructions. B cell purity was consistently >90% based on B220 or CD19 staining. Enriched B cells were cultured at 5×10^6^ cells/ml for 8–72 h in the presence of 4 ng/ml recombinant BAFF (R&D Systems) to increase B cell survival [22]. This low amount of BAFF was added to increase B cell survival, and did not change the parameters evaluated for B cell activation (data not shown). Where appropriate, cells were treated with 2 or 10 µg/ml F(ab′)_2_ goat anti mouse IgM antibodies (Jackson ImmunoResearch Laboratories), 20 µg/ml LPS, 2 µM cycloheximide, 0.1 µM actinomycin D in 0.4% dimethyl sulfoxide (DMSO), 0.2 µM cyclosporin A in 0.2% DMSO (all from Sigma-Aldrich), 15 µg/ml anti-CD40 antibodies (FGK 45.5), 50 ng/ml recombinant mouse IL-4 (eBioscience), 100 and 1000 U/ml IFNγ (R&D Systems), 100 and 1000 U/ml IFNα (PBL InterferonSource) or 1 µg/ml GITRL. The GITRL reagent consisted of a synthetic His-GITRL protein and anti-polyHistidine antibodies used at 10 µg/ml (R&D Systems). In cell cultures with treatments dissolved in DMSO, DMSO was added to control wells at the same concentration.

For cell proliferation, splenic cells were purified over Ficoll-Paque Premium (GE Healthcare) and labeled with 2.5 µM Carboxyfluorescein Succinimidyl Ester (CFSE; Invitrogen Molecular Probes). CFSE-labeled cells were cultured for four days in the presence of 4 ng/ml BAFF. Additional treatments included 10 µg/ml anti-IgM antibodies, 15 µg/ml anti-CD40 antibodies (1C10), and a combination of anti-IgM and anti-CD40 antibodies or anti-IgM antibodies and 50 ng/ml IL-4. Agonistic anti-GITR DTA-1 antibodies (from eBioscience or produced in house) were added to some cultures at 10 µg/ml.

### Immunizations and ELISA

Mice were immunized i.p. with 100 µg NP_36_-CGG (4-Hydroxy-3-nitrophenylacetic hapten conjugated to chicken gamma globulin, Biosearch Technologies) mixed at equal volume with Alu-Gel-S (Aluminum hydroxide, SERVA) in PBS, or with 5 µg NP_28_-FICOLL (4-Hydroxy-3-nitrophenylacetic hapten conjugated to AminoEthylCarboxyMethyl-FICOLL, Biosearch Technologies) in PBS. For NP-CGG boost immunizations, mice were injected with 5 µg NP_36_-CGG in PBS 178 days after the first immunization.

Total serum Ig isotype levels were measured by coating 96-well Nunc-Immuno MaxiSorp plates (Thermo Fisher Scientific) with 5 µg/ml of goat anti-mouse IgM, IgG1, IgG2b, IgG2a (SouthernBiotech) or IgE antibodies (R35-72, BD Pharmingen). After blocking with PBS, 1% BSA, diluted serum samples were added and further serially diluted. Standard curves were generated by serially diluting purified mouse IgM, IgG1, IgG2b, IgG2a (SouthernBiotech) and IgE (BD Pharmingen). Isotype-specific antibodies were detected using alkaline phosphatase (AP)-conjugated goat anti-mouse IgM, IgG1, IgG2b, IgG2a and rat anti-mouse IgE (SouthernBiotech) antibodies, and developed by the addition of AP substrate (p-nitrophenyl phosphate, Sigma).

NP-specific antibodies were measured as previously described [23]. Briefly, plates were coated with 2 µg/ml NIP_22_-BSA (Biosearch Technologies). After blocking as described above, serum samples were added and serially diluted. Isotype-specific antibodies were detected with isotype-specific AP-conjugated goat anti-mouse Ig antibodies (SouthernBiotech) and developed with AP substrate. Standard curves were generated using NP-specific mouse monoclonal antibodies: B1-8μ (NP-specific IgM), S24/63/63 (NP-specific IgG3), N1G9 (NP-specific IgG1), D3-13F1 (NP-specific IgG2b) and S43-10 (NP-specific IgG2c/a).

The absorbance values of plates were read at 405 nm on a Versamax ELISA reader (Molecular Devices), and the data were analyzed with SoftMax Pro 5 Software (Molecular Devices).

### 5-bromo-2′-deoxyuridine (BrdU) incorporation

Mice were fed 0.8 mg/ml BrdU (Sigma) in water containing 1% D-glucose *ad libitum* for one or two weeks. BrdU-containing water was changed twice a week. BrdU incorporation in B cell subsets was assessed by staining cells for appropriate surface markers and then for BrdU using the APC BrdU Flow Kit (BD Pharmingen) according to manufacturer's instructions.

### Generation of mixed bone marrow chimeras

Donor bone marrow cells from 129S1, GITR^−/−^ or B6.SJL (Ly5.1) mice were depleted of CD49b^+^, CD3^+^ and IgM^+^ cells by staining with a cocktail of biotinylated anti-CD49 (DX5), anti-CD3 (145-2C11) and anti-IgM (R33-24) antibodies, followed by anti-biotin magnetic beads. Cells bearing those markers were removed by AutoMACS (Miltenyi). Cell purity was determined to be ≥97% by flow cytometric analysis of CD49, CD3, and IgM expression. After cell depletion, 129S1 and GITR^−/−^ donor cells were mixed at 1∶3 ratio with B6.SJL donor cells and injected i.v. (1–2×10^6^ cells/mouse) into irradiated C57BL/6J recipient mice as previously described [24]. Bone marrow chimeras were sacrificed and organs harvested after 8 weeks, and B cell development and maturation was assessed by flow cytometry.

### Statistical data analysis

Data were analyzed using GraphPad Prism software. Statistical significance was assessed with a one-tailed, unpaired Student's *t* test. P<0.05 was considered significant. Data are represented as means ± standard deviation (SD) or geometric means ±SEM.
